# Liner dissociation in a large-diameter ceramic-bearing acetabular component: a report of five cases

**DOI:** 10.1186/s12891-022-05082-6

**Published:** 2022-02-09

**Authors:** Lazaros Kostretzis, Sagi Martinov, Martin Lavigne, Vincent Massé, Pascal-André Vendittoli

**Affiliations:** 1 Surgery Department, Hôpital Maisonneuve-Rosemont, Montreal University, 5415 Boulevard de l’Assomption, Montréal, Québec H1T 2M4 Canada; 2 Clinique Orthopédique Duval, Laval, QC Canada

**Keywords:** Total hip arthroplasty, Ceramic liner, Dissociation, Monoblock acetabular component

## Abstract

**Background:**

Ceramic-on-ceramic (CoC) bearings for total hip arthroplasty (THA) have been offering very favorable results and survivorship since their introduction. In order to increase range of movement (ROM) and decrease dislocation rates, some manufacturers have introduced larger diameter head (LDH) CoC bearings. This has been achieved with the use of preassembled cup designs, in which the ceramic liner is already fitted into the metal backing and implanted as a monoblock component by the surgeon. In this report we present data from a series of 5 patients with ceramic liner dissociation from a monoblock cup.

**Case presentation:**

All cases were overweight men with acetabular components of 56 or 58 mm. After a mean of 5.5 (range, 3.5-6.7) years, all patients reported sudden pain and audible noise when performing activities of daily living. Liner displacement was suspected on plain radiographs and confirmed by Ct-scan. Pneumarthrosis was present in all cases. Taper modular junction wear and corrosion signs were observed in the four revised patients.

**Conclusion:**

Although one of our case is still treated conservatively, implant revision is probably inevitable. Further LDH CoC implant design should take in consideration this potential complication by avoiding bearing diameters over 40mm and/or improving locking mechanism or by providing a real monoblock acetabular implant.

## Background

Ceramic-on-ceramic (CoC) bearings for total hip arthroplasty (THA) were introduced in the 1970s in France [1], offering very favorable results and survivorship [2]. Modern ceramic has enabled minimization of clinical fractures [3] and introduction of larger diameter head (LDH) (more than 36mm) CoC bearings in order to increase range of movement (ROM) and decrease dislocation rates, while avoiding the associated problems seen in metal-on-metal (MoM) bearings. The latter was achieved with the use of preassembled cup designs, in which the ceramic liner is already fitted into the metal backing by the manufacturer and implanted as a monoblock component by the surgeon. In the last years, LDH CoC designs were available from Zimmer Biomet (Maxera™), Depuy Synthes (DELTAMOTION®) and Adler Ortho (AGILIS Ti-Por®). Short- and mid- term clinical data although encouraging is still very limited [2, 4–7].

Despite promising results with LDH CoC THA, potential problems specific to the implant design may still arise. The incidence of noise generation has been reported to range from 3.5% to 36% [2, 4–7], depending highly on the method of data acquisition. Groin pain has been reported to be present in 7.1% of these patients [8]. Although not reported, fracture of the ceramic liner is still a potential complication.

Since 2011, we performed in our academic centre, 3047 LDH CoC THAs using the Maxera hip system (Zimmer Biomet, Warsaw, Not for sale in the USA and Japan). This report presents data from a series of 5 patients with ceramic liner dissociation. To the best of our knowledge, this complication has not been previously reported. This study was approved by our institutional review board. All of the patients were informed that data concerning their cases would be submitted for publication, and they provided consent.

## Case presentation

### Patient 1

**Fig. 1:**
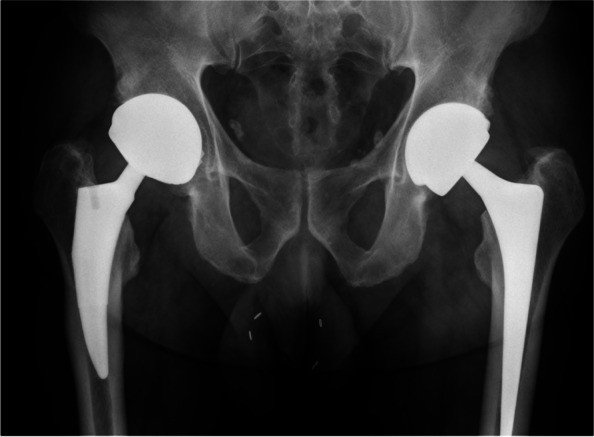
Postoperative anteroposterior radiograph after implantation of the left THA

**Fig. 2:**
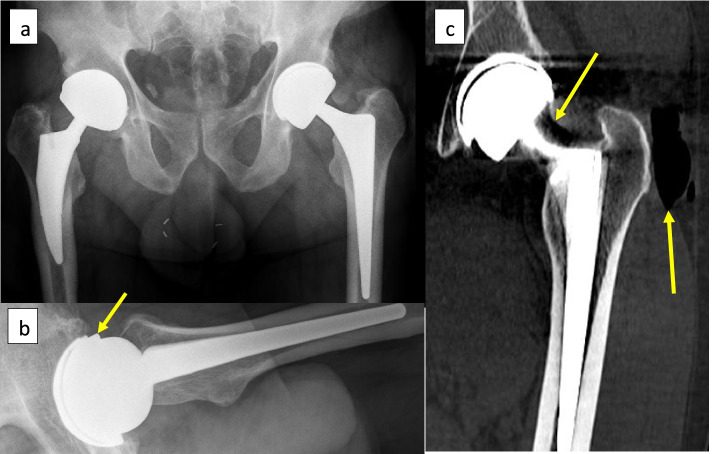
**a** Anteroposterior pelvic revealing a thin crescent shaped radiolucency between the acetabular shell and the ceramic liner and pneumarthrosis. **b** Lateral hip radiograph with the arrow pointing at the displaced liner. **c** CT coronal view of the left hip demonstrating the thin crescent shaped radiolucency between the acetabular shell and the ceramic liner, 2 arrows are pointing at intra-articular gas and gas around the greater trochanter

**Fig. 3:**
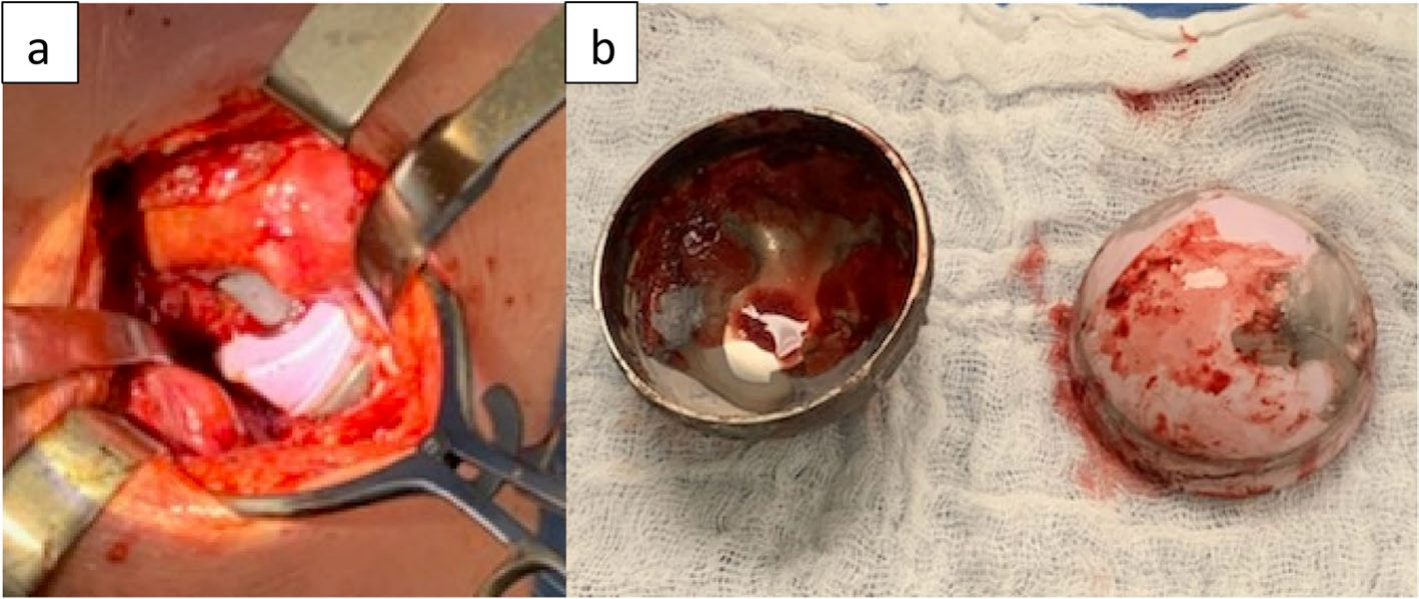
**a** Intra operative image showing the dissociated liner (mobile). **b** The acetabular shell and the ceramic liner showing important signs of wear and corrosion on the taper surfaces

**Fig. 4:**
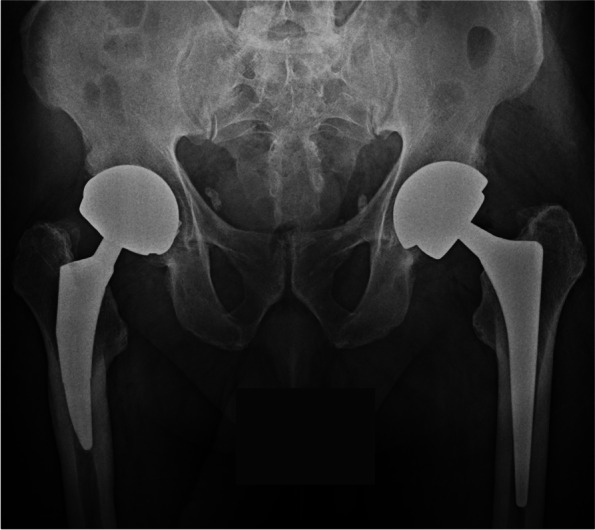
Anteroposterior pelvic radiographs after revision of the left acetabular component with a new Maxera component 4mm larger (bearing 48 mm)

A patient with primary osteoarthritis was treated at the age of 56 and 59 with a right LDH MoM THA and a left LDH CoC THA, respectively (BMI: 32.8, cup diameter: 58 mm, Fig. 1, Table 1). Seventy-eight months after the left THA the patient reported sharp hip pain. No trauma had occurred. Ceramic insert separation and pneumarthrosis were observed on x-ray and confirmed by CT scan (Fig. 2a, b and c). The patient was re-operated two weeks later. The revision surgery revealed a wide-open posterior capsule with a large collection of yellowish fluid. Ceramic liner was clearly displaced and mobile (Fig. 3a). After using an adapted extraction tool with minimal bone loss, a new Maxera cup, 4 mm larger than in the index one was implanted (Fig. 4). Titanium shell showed wear signs and titanium debris (Fig. 3b). The patient followed standard post-operative rehabilitation. No complication has been observed at 8 months follow-up.

**Table 1:** Summary of patients’ characteristics and related findings

Patient number	Gender	Age at index procedure (years)	BMI (kg/m^**2**^)	Cup size/head diameter	Cup inclination/anteversion angles on CT evaluation	Time to dissociation diagnosis (months)	Sudden noise	Sudden pain	Pneumarthrosis on radiographic evaluation	Revision surgery	Joint fluid at revision surgery	Soft tissue changes/signs of ARMD
1	Male	59	32.8	58 mm/48 mm	42°/16°	78	Yes	Yes	Yes	Yes	Large amount	Yes
2	Male	49	34.2	58 mm/48 mm	44°/7°	42	Yes	Yes	Yes	Yes	Minimal	No
3	Male	55	46.2	56 mm/44 mm	38°/27°	60	Yes	Yes	Yes	No after 1 year	-	-
4	Male	39	41.5	58 mm/48 mm	45°/9°	69	Yes	Yes	Yes	Yes	Minimal	No
5	Male	53	27.4	56 mm/44 mm	39°/25°	80	Yes	Yes	Yes	Yes	Large amount	Yes

### Patient 2

A 49-year-old male with severe symptomatic primary osteoarthritis was treated by simultaneous bilateral CoC LDH THA (BMI: 34.2, cup diameter: 58 mm, Table 1). The patient reported occasional noise in his articulation on follow-up but was otherwise very satisfied and very active. After 44 months, during a hockey game, while resting on the bench, the patient heard a loud articular noise on the left side and felt a “shock” in his limb and acute pain. He reported no trauma during the game. The AP x-ray showed a small gas collection laterally to the acetabulum. The CT confirmed the suspicion of ceramic liner displacement. Two months later, pneumarthrosis was resorbed on follow-up X-ray and the patient was now asymptomatic. Nonetheless, it was decided to perform a THA exploration/revision. During revision surgery, the capsule scar tissue did not show any abnormality. A small amount of intra-articular transparent liquid was found after the capsulotomy. The ceramic liner was slightly displaced in the metal cup but stable. After using an adapted extraction tool, the acetabular cavity showed a minimal bone loss and a 2 mm larger diameter Maxera cup was implanted. At one-year post revision follow-up, his recovery has been uneventful, with no pain or articular noise.

### Patient 3

**Fig. 5:**
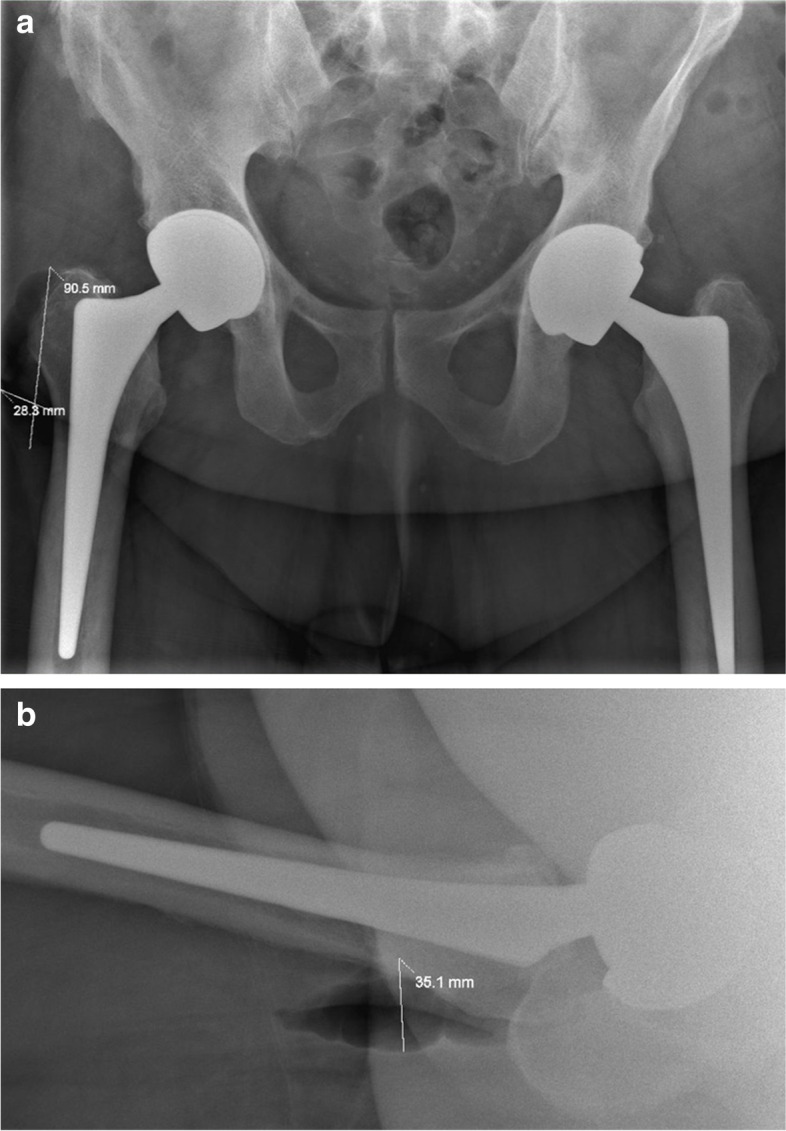
AP pelvis (**a**) and lateral (**b**) radiographs showing periarticular gas and suspicion of ceramic liner disimpaction on the right THA

**Fig. 6:**
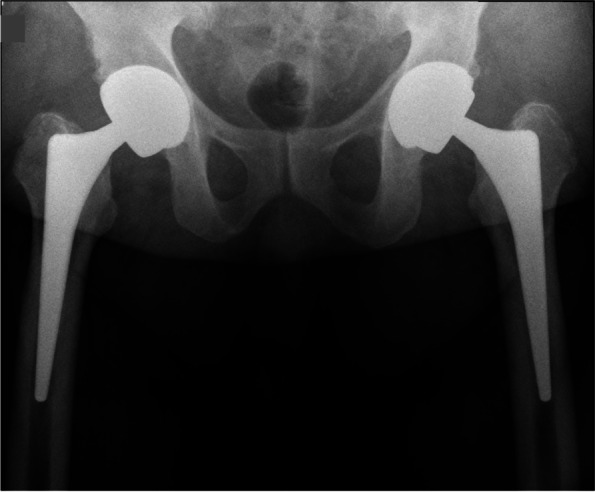
AP pelvis radiograph, 12 months after the liner dissociation on his right THA. Patient is still been pain free and prefers to pursue a conservative treatment

A 55-year-old male with primary osteoarthritis was treated by simultaneous bilateral CoC LDH THA (BMI: 46.2, cup diameter: 56 mm, Table 1). The patient had a standard and uneventful rehabilitation. At 5-year follow-up the patient reported a sudden hip pain while walking. No concurrent trauma was reported; however, the patient had fallen twice three years after the arthroplasty. Following the fall, the patient did not seek medical advice, since no symptoms were present. The patient was admitted to the emergency unit of another hospital where plain radiographs showed pneumarthrosis and suspicion of ceramic liner disimpaction (Fig. 5a and b). He then came back to our institution 2 weeks later with minimal pain. It was decided to follow a conservative treatment. After 12 months, patient has been pain-free and prefers to pursue a conservative treatment (Fig. 6).

### Patient 4

A 39-year-old with left femoral head avascular necrosis was treated by CoC LDH THA (BMI: 41.5, cup diameter: 58 mm, Table 1). Sixty-nine months after THA, the patient had abrupt pain in the left groin and buttock area after bending forward. No history of trauma was reported by the patient. Millimetric separation of the ceramic insert and pneumarthrosis was observed on x-ray and confirmed by CT scan. The patient was operated two weeks later. After arthrotomy of a well closed articular capsule, we found a small amount of transparent liquid without any signs of metallosis. The ceramic liner was misaligned, however after the extraction of the acetabular component it was impossible to separate the ceramic liner from the titanium shell. A Maxera cup 4 mm larger than in the index THA was implanted. The patient followed a standard post-operative rehabilitation without any adverse events.

### Patient 5

**Fig. 7:**
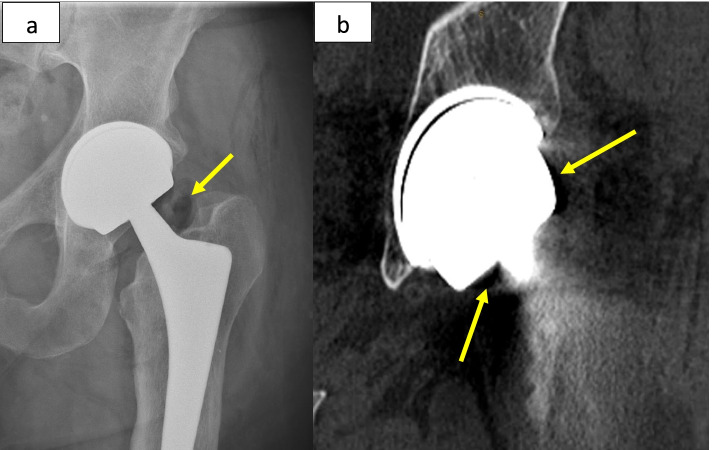
**a** Anteroposterior left hip revealing a thin crescent shaped radiolucency between the acetabular shell and the ceramic liner and pneumarthrosis (arrow). **b** CT-scan view of the left hip demonstrating the thin crescent shaped radiolucency between the acetabular shell and the ceramic liner, 2 arrows are pointing at the intra-articular gas

**Fig. 8:**
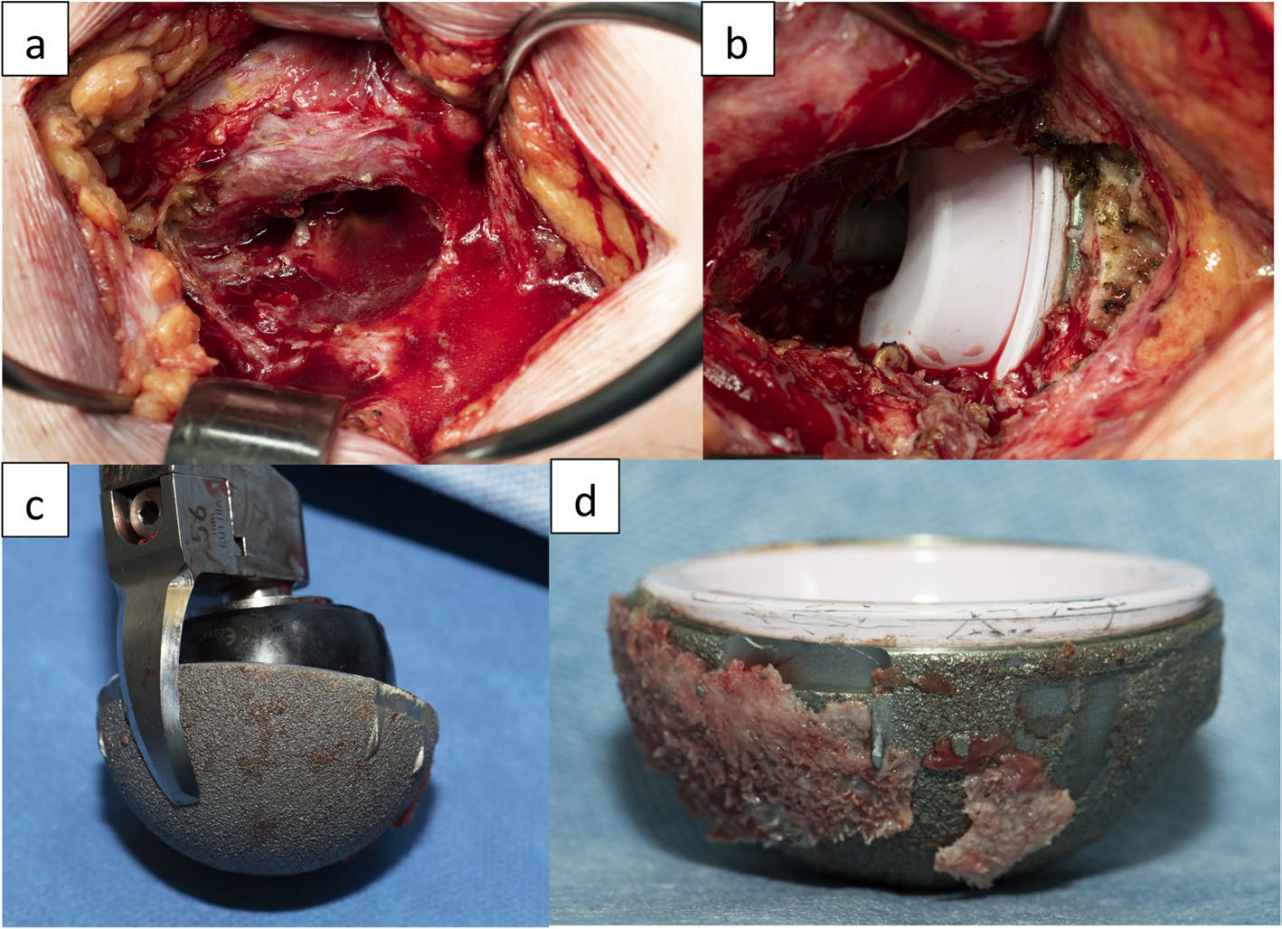
**a** Revision surgery revealed a partially open posterior capsule with a large collection of yellowish fluid. **b** Intra-operative confirmation of the misaligned ceramic insert. **c** A curved blade explantation system was used to remove the implant with minimal bone loss. **d** The liner inside the explanted cup was misaligned but not mobile. We had to knock multiple times on the side of the cup to disengage the liner

**Fig. 9:**
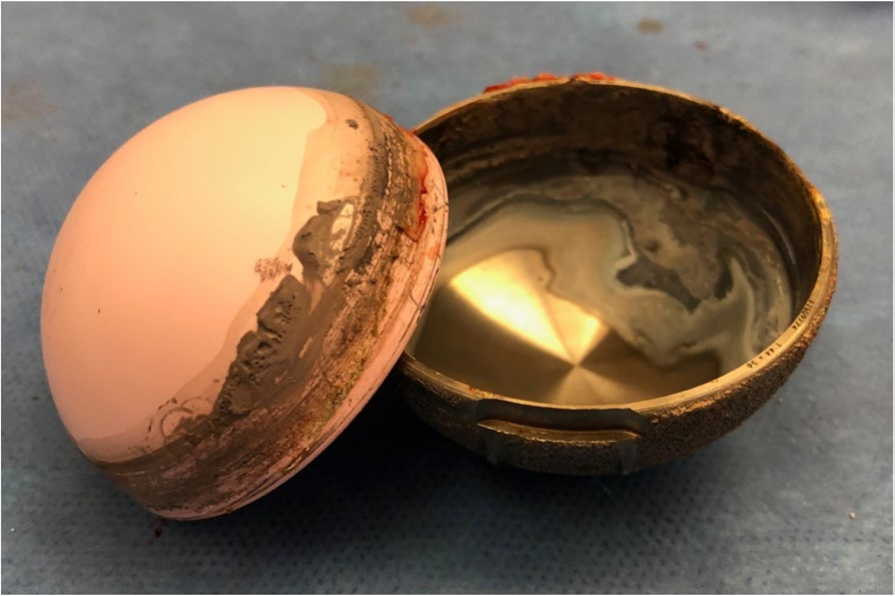
The acetabular shell and the ceramic liner taken apart and showing important signs of wear and corrosion debris on the taper surfaces

**Fig. 10:**
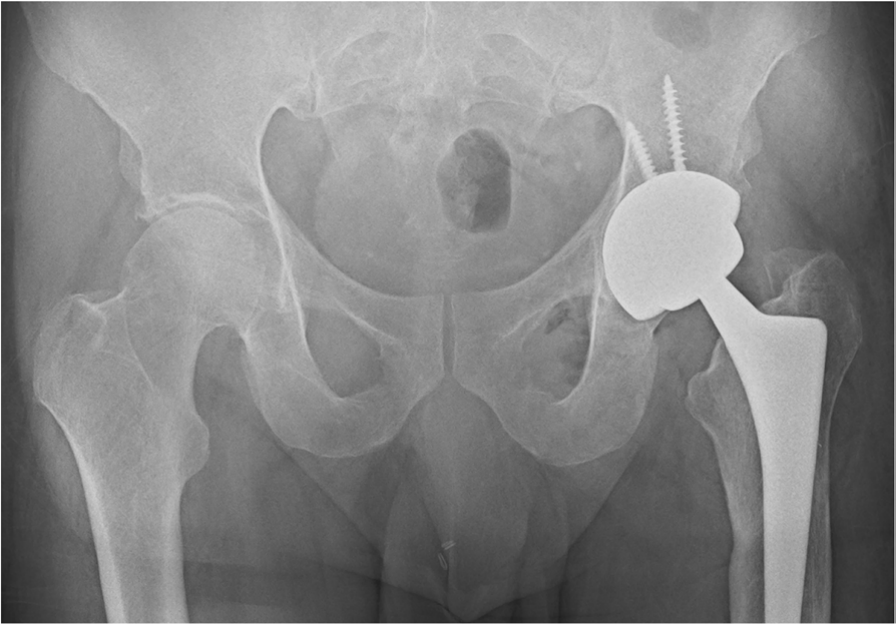
Post-operative pelvis radiograph showing the revised acetabular component 2 mm larger than the explanted Maxera cup. Modular cup with ceramic acetabular bearing (40 mm) and supplemental fixation with 2 screws

A 53-year-old male with primary OA underwent left CoC LDH THA (BMI:27.4, cup diameter: 56mm, Table 1). Eighty months after an active and uneventful post-operative period, while opening a sliding window the patient felt a clunk in his hip followed by severe pain, making him unable to walk. The patient was admitted to the regional hospital where the x-ray and CT scan demonstrated an intra-articular fluid, gas collection and ceramic liner dissociation (Fig. 7a and b). The patient was re-operated four weeks later (Fig. 8a-d). The revision surgery revealed a partially open posterior capsule with a large collection of yellowish fluid. The ceramic liner was misaligned but not mobile. Upon disimpaction of the Maxera ceramic liner on the side table, the implants modular junctions showed important wear with Ti debris (Fig. 9). With minimal bone loss during explantation, a new modular cup 2mm larger than in the index one was implanted with 2 screws and a 40mm ceramic insert was impacted (Fig. 10).

## Discussion and conclusions

Even though intra-operative ceramic liner malseating [9–11] has been reported, to our knowledge, this article is the first description of spontaneous post-operative ceramic liner dissociation. The five reported cases had an LDH CoC Maxera implant, where the liner is inserted during manufacturing process and implanted by the surgeon as a monobloc implant. The insertion of the acetabular component by all the surgeons was performed according to the surgical technique suggested by the manufacturer. All patients in our report were active males with a mean BMI of 36.4 (range, 27.4 - 46.2) and had well-functioning THAs before ceramic liner dissociation. No patient reported significant trauma or injury before the event. All patients characteristically reported an audible incident, sudden discomfort and pain. The mean time in situ was 66 months (range, 42–80). On CT evaluation, all cups were considered well positioned with a mean inclination of 42° (range, 38°–45°) and a mean anteversion of 17° (range, 7° -27°). Intra-operative inspection of all dissociated liners demonstrated shearing of the peripheral locking tabs, with no signs of ceramic fracture. Component diameters were 58 mm (bearing 48 mm) in three cases and 56 mm (bearing 44 mm) in two.

Acetabular component modularity was introduced in THA to allow bearing exchange in case of premature polyethylene wear and to simplify implant inventory. In metal backed MoP acetabular component, early postoperative polyethylene liner dissociations have been attributed to intra-operative liner malseating or poor locking mechanism design while late dissociations are believed to be associated with femoral neck impingement against the liner or edge loading causing liner micro motion and liner locking mechanism wear [12–14]. An important factor in polyethylene liner dissociation is the suboptimal locking mechanism of multi-bearing acetabular shells implant design. Multi-bearing acetabular components require an inner tapering angle that couples metal ceramic liners generating limited static friction and suboptimal for a polyethylene insert [15].

Regarding CoC THA, intra-operative ceramic liner malseatings were reported by different authors [9–11]. Because a malseated liner could increase the risk of liner fracture, squeaking, and titanium fretting corrosion, recommendation is to revise these cases when identified on post-operative radiographs [10]. On the other hand, spontaneous post-operative ceramic liner dissociation has not been reported. Ceramic modular junction design requires a friction force across the titanium and ceramic interfaces to keep the component assembled. Ideal ceramic-titanium taper angle should be 18° over smaller angles [16]. As the titanium is thinner and has a lower modulus of elasticity than ceramic, the shell will expand during ceramic liner impaction and act as an elastic band creating compressive stress on the ceramic liner. For intraoperative manual assembly, meticulous surgical technique was shown essential. Surgeon should make sure to have clean implants (no blood, fat or debris), precise component alignment and apply sufficient impaction force. In modular implant, press fitted in stiff bone can cause shell deformation of thin titanium shell, affecting its locking mechanism and cause liner malseating or chipping.

All cases of liner dissociation presented in this report are LDH THA with the Maxera acetabular component. To prevent the cited above potential problems, the BIOLOX delta taper liner is pre-assembled and secured into the titanium shell. It is then implanted by the surgeon as a monobloc component. This component has an optimal locking mechanism design for ceramic with a 18° taper angle. The component is hemispherical and available in outer diameters from 42 to 66 mm. The shell substrate is manufactured from Tivanium Ti-6Al-4V alloy with a thin layer of titanium vacuum plasma spray coating (Ti-VPS) applied to the exterior of the shell. The BIOLOX delta liner articulates with a BIOLOX delta femoral head of a 32 to 48 mm diameter, that matches the inner diameter of the liner. Each bearing diameter matches specific cup diameters (Table 2). The metallic shell thickness being constant, larger sizes for the same bearing diameter have a thicker ceramic liner. The cup is press-fitted into a 2mm-smaller reamed hemispherical acetabular host-bone. In our 5 cases, cup diameters were exclusively 56 mm and 58 mm (respective bearing diameters of 44 and 48 mm). With 3047 Maxera implantation over the last 10 years in our institution, the overall liner dissociation is 0.16% (5 in 3047). The total percentage of 56mm cups used is 17.6% (*N *= 536) and 12.7% (*N* = 388) for 58mm. The respective relative rates for 56mm and 58mm cups are 0.37% and 0.77% (respectively 2.3 and 4.8 time the overall rate). No case was encountered with 36 or 40 mm bearings (≤54 cups).

**Table 2:** Matching of acetabular component and femoral head size for Maxera cup (Zimmer, Warsaw)

**Acetabular size (mm)**	46	48	50	52	54	56	58
**Femoral head size (mm)**	36	36	40	40	44	44	48

As mentioned above, modular acetabular metallic shell can deform under press fit implantation. Such deformation was also reported with metal-on-metal (MoM) LDH one-piece acetabular constructs. This deformation is greater with increased bone stiffness and larger cups with thinner walls [17, 18]. Our five cases were males with high BMI, hard bone and large components. Titanium cup deformation during impaction could have deformed the locking mechanism and lead to subsequent micromotion. On the other hand, preassembled ceramic liner of the Maxera cup increases significantly the construct stiffness and should minimize metal shell deformation. A cadaveric study by Beckmann et al. [19] has demonstrated elastic deformation at the acetabular rim, during cyclic loading that replicates the limited loading of normal gait as in the early postoperative period. The diametrical change of the component rim during cyclical loading could cause micromotion in the taper locking mechanism of the cup and liner, which in turn might cause wear, taper damage and subsequent liner dissociation.

**Fig. 11:**
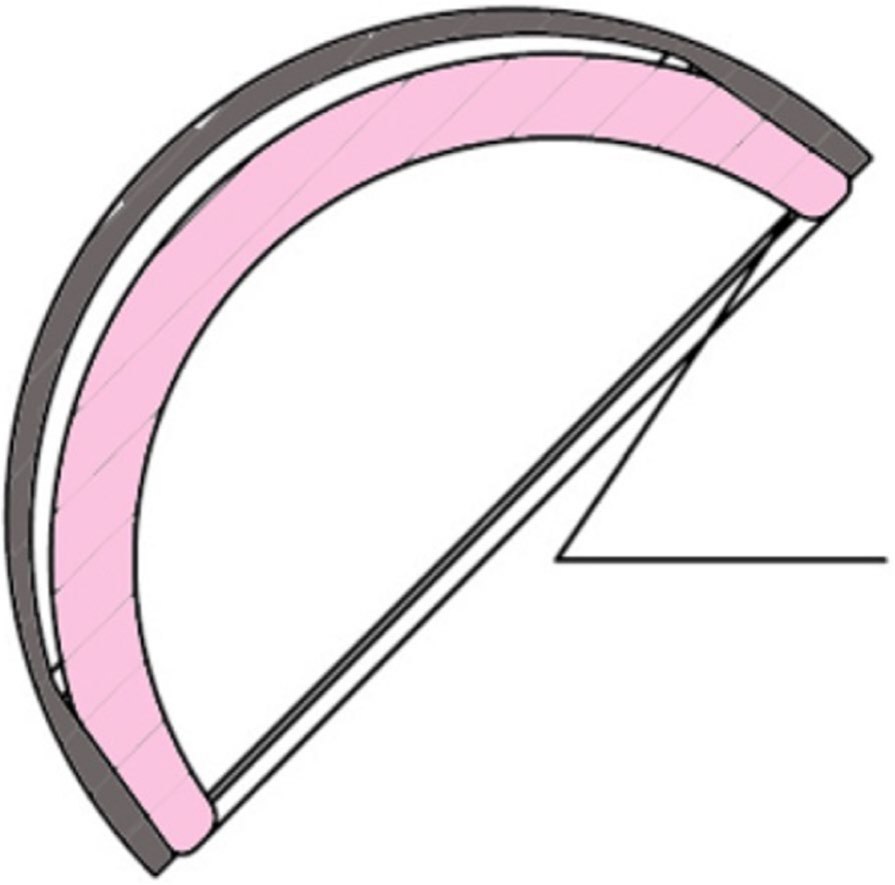
A small space between the dome of the ceramic liner and the titanium cup exists to allow better taper interference

Whatever the cause of taper disfunction, chronic micromotion of the rigid ceramic liner over time can remove the passivation layer of the titanium taper surface and lead to fretting corrosion [20], with subsequent release of Ti particles as observed in the patients we tested for Ti levels. We observed in all our cases pneumarthrosis. Weber et al. [20] has reported six cases of post-THA pneumarthrosis caused by diatomic hydrogen accumulation in modular Ti/Ti femoral stems. The proposed mechanism is cyclic motion that causes abrasion of the external surface of the implant which releases Ti ions that are oxidized when exposed to joint fluid which, in turn, create hydrogen ions. In their cases, mean serum Ti levels were reported to be elevated (33 μg/L). Although the ceramic-titanium interface is different from the Ti/Ti one, the hydrogen accumulation phenomenon could be similar. These levels are much higher than previously published Ti levels ranging between 1.2 and 1.4 in 57 well-functioning Maxera LDH THA cases after 79 months mean follow-up [21]. Because the pneumarthrosis disappeared in the following weeks after the liner dissociation, we believe that the intra-articular air observed was trapped in the space between the metallic shell and ceramic liner until liner dissociation. As shown in Fig. 11, to allow better taper interference, the dome of the ceramic liner does not contact the floor of the titanium shell, leaving a small space between the ceramic liner and the titanium cup. For this reason, a small lucent line may be visible in totally normal cup and should not be interpreted as a liner disengagement. Gas trapped into that space may also create a positive pressure and play a role in the liner dislodgment.

In this study, we report five ceramic liner dissociations of a pre-assembled LDH THA titanium shell. These cases had specific clinical presentations including sudden pain, audible “clunk” noise, and the radiographic presence of a wider crescent shaped radiolucency between the shell and the liner, pneumarthrosis and subtle sign of liner displacement. Although one of our case is still treated conservatively, implant revision is probably inevitable. For the revision surgery, the authors recommend using a larger diameter cup of the same monoblock ceramic implant or a modular ceramic cup that allows insertion of screws, when additional stability is needed. Since, this complication is fairly new the authors still use and recommend this implant but are more vigilant in the follow up visits of patients with any of the aforementioned clinical findings. Further LDH CoC implant design should take in consideration this potential complication in the largest bearings sizes by avoiding bearing diameters over 40mm and/or improving locking mechanism or by providing a real monoblock acetabular implant.

## Data Availability

The datasets used and analyzed during the current study are available from the corresponding author on reasonable request.

## Abbreviations

ARMD: Adverse reaction to metal debris
CoC: Ceramic-on-ceramic
CT: Computer tomography
LDH: Large diameter head
MoM: Metal-on-metal
ROM: Range of movement
THA: Total hip arthroplasty

## References

[1] Boutin P (2014). Total arthroplasty of the hip by fritted alumina prosthesis. Experimental study and 1st clinical applications. Orthop Traumatol Surg Res..

[2] Vendittoli PA, Shahin M, Rivière C, Barry J, Lavoie P, Duval N (2021). Ceramic-on-ceramic total hip arthroplasty is superior to metal-on-conventional polyethylene at 20-year follow-up: a randomised clinical trial. Orthop Traumatol Surg Res..

[3] Howard DP, Wall PDH, Fernandez MA, Parsons H, Howard PW (2017). Ceramic-on-ceramic bearing fractures in total hip arthroplasty. Bone Joint J..

[4] McDonnell SM, Boyce G, Baré J, Young D, Shimmin AJ (2013). The incidence of noise generation arising from the large-diameter Delta Motion ceramic total hip bearing. Bone Joint J..

[5] Tai SM, Munir S, Walter WL, Pearce SJ, Walter WK, Zicat BA (2015). Squeaking in large diameter ceramic-on-ceramic bearings in total hip arthroplasty. J Arthroplasty..

[6] Goldhofer MI, Munir S, Levy YD, Walter WK, Zicat B, Walter WL (2018). Increase in benign squeaking rate at five-year follow-up: results of a large diameter ceramic-on-ceramic bearing in total hip arthroplasty. J Arthroplasty..

[7] Deny A, Barry J, Hutt JRB, Lavigne M, Massé V, Vendittoli PA (2018). Effect of sleeved ceramic femoral heads on titanium ion release. Hip Int..

[8] Lavigne M, Vendittoli PA, Virolainen P (2020). Large head ceramic-on-ceramic bearing in primary total hip arthroplasty: average 3-year follow-up of a multicentre study. Hip Int..

[9] Shnaekel AW, Mayes WH, Stambough JB, Edwards PK, Mears SC, Barnes CL (2020). Dissociation of acetabular polyethylene liners with a Morse taper design. J Arthroplasty.

[10] Napier RJ, Diamond O, O’Neill CKJ, O’Brien S, Beverland DE (2017). The incidence of dissociated liners in 4,751 consecutive total hip arthroplasties using Pinnacle polyethylene acetabular liners. Hip Int..

[11] Yun A, Koli EN, Moreland J (2016). Polyethylene liner dissociation is a complication of the DePuy pinnacle cup: a report of 23 cases. Clin Orthop Relat Res.

[12] Grazette AJ, Foote J, Whitehouse MR, Blom AW (2016). A review of outcomes and modes of presentation following liner dissociation from Harris-Galante uncemented acetabular components. Hip Int.

[13] Langdown AJ, Pickard RJ, Hobbs CM, Clarke HJ, Dalton DJN, Grover ML (2007). Incomplete seating of the liner with the Trident acetabular system: A cause for concern?. J Bone Joint Surg Br..

[14] Jaeger S, Uhler M, Schroeder S, Beckmann NA, Braun S (2020). Comparison of different locking mechanisms in total hip arthroplasty: relative motion between cup and inlay. Materials (Basel)..

[15] Lee YK, Kim KC, Jo WL, Ha YC, Parvizi J, Koo KH (2017). Effect of inner taper angle of acetabular metal shell on the malseating and dissociation force of ceramic liner. J Arthroplasty.

[16] Lee YK, Lim JY, Ha YC, Kim TY, Jung WH, Koo KH (2021). Preventing ceramic liner fracture after Delta ceramic-on-ceramic total hip arthroplasty. Arch Orthop Trauma Surg..

[17] Springer BD, Habet NA, Griffin WL, Nanson CJ, Davies MA (2012). Deformation of 1-piece metal acetabular components. J Arthroplasty.

[18] Squire M, Griffin WL, Mason JB, Peindl RD, Odum S (2006). Acetabular component deformation with press-fit fixation. J Arthroplasty..

[19] Beckmann NA, Bitsch RG, Bormann T, Braun S, Jaeger S (2019). Titanium acetabular component deformation under cyclic loading. Materials (Basel)..

[20] Weber AE, Skendzel JG, Waxman DL, Blaha JD (2013). Symptomatic aseptic hydrogen pneumarthrosis as a sign of crevice corrosion following total hip arthroplasty with a modular neck. JBJS Case Connect.

[21] Eichler D, Barry J, Lavigne M, Massé V, Vendittoli PA (2021). No radiological and biological sign of trunnionosis with large diameter head ceramic bearing total hip arthroplasty after 5 years. Orthop Traumatol Surg Res.

